# Pilot Study of a Novel Device for Endoscopic Vacuum Therapy for Anastomotic Leak

**DOI:** 10.14309/crj.0000000000002141

**Published:** 2026-05-26

**Authors:** Maria A. Cassera, Liam Hilson, Anand Baxi, Anand Singla, Michael Saunders, Eric Seibel, Adam W. Templeton

**Affiliations:** 1 Division of Gastroenterology, University of Washington Medical Center, Seattle, WA; 2 Department of Mechanical Engineering, University of Washington, Seattle, WA

**Keywords:** endoscopic vacuum therapy, esophageal perforation, gastrointestinal leak

## Abstract

Endoscopic vacuum-assisted therapy (EVT) is an emerging technology using wound vacuum-assisted closure systems for gastrointestinal leaks. We present the case of 65-year-old man with esophageal adenocarcinoma who underwent laparoscopic transhiatal esophagogastrostomy, complicated by an anastomotic leak. This is the first case of a novel one-piece EVT device to evaluate the safety and efficacy in treatment of gastrointestinal leaks. Our case demonstrates that this novel EVT device is both safe and effective, with the added benefit of a pre-assembled design that reduces procedural complexity and may facilitate wider adoption of EVT for gastrointestinal leaks.

## INTRODUCTION

Gastrointestinal leaks result from perforation along the interior wall of the gastrointestinal tract following surgery, iatrogenic perforations, and spontaneous perforations such as Boerhaave syndrome. Historically, treatment of gastrointestinal leaks located in the esophagus have included surgical repair or placement of self-expanding metal stents; however, these methods are associated with significant morbidity.

**Figure 1.**
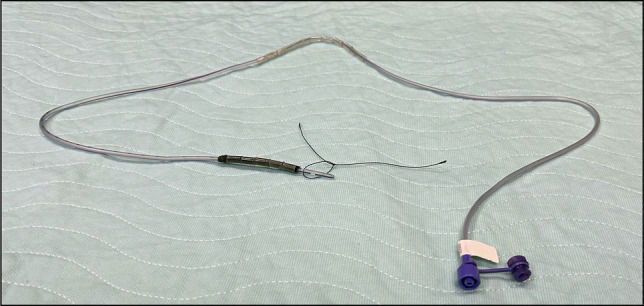
Novel endoscopic vacuum-assisted therapy device.

Endoscopic vacuum-assisted therapy (EVT) has emerged as an effective option for treating gastrointestinal leaks; however, widespread adoption has been challenged by the high level of skill required for placement and the technical complexity of device assembly. To mitigate these challenges, we have developed a novel one-piece EVT device consisting of a compressed pellet sponge and tube kit, and features a sponge encased in a dissolvable shell which expands upon positioning at the leak site (Figure 1).^1^ We present the first case of this novel EVT device to evaluate the safety and efficacy in treatment of gastrointestinal leaks.

## CASE REPORT

**Figure 2.**
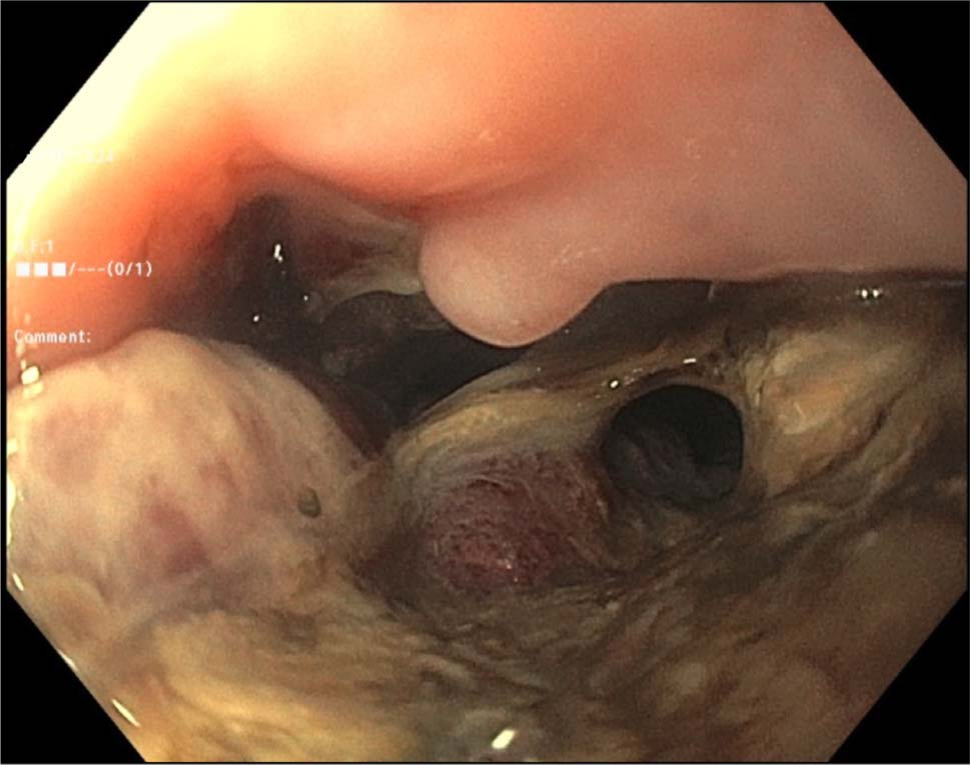
Upper endoscopy revealed an esophagogastric anastomosis characterized by ulceration, an intact staple line, and an adjacent 9 mm defect.

**Figure 3.**
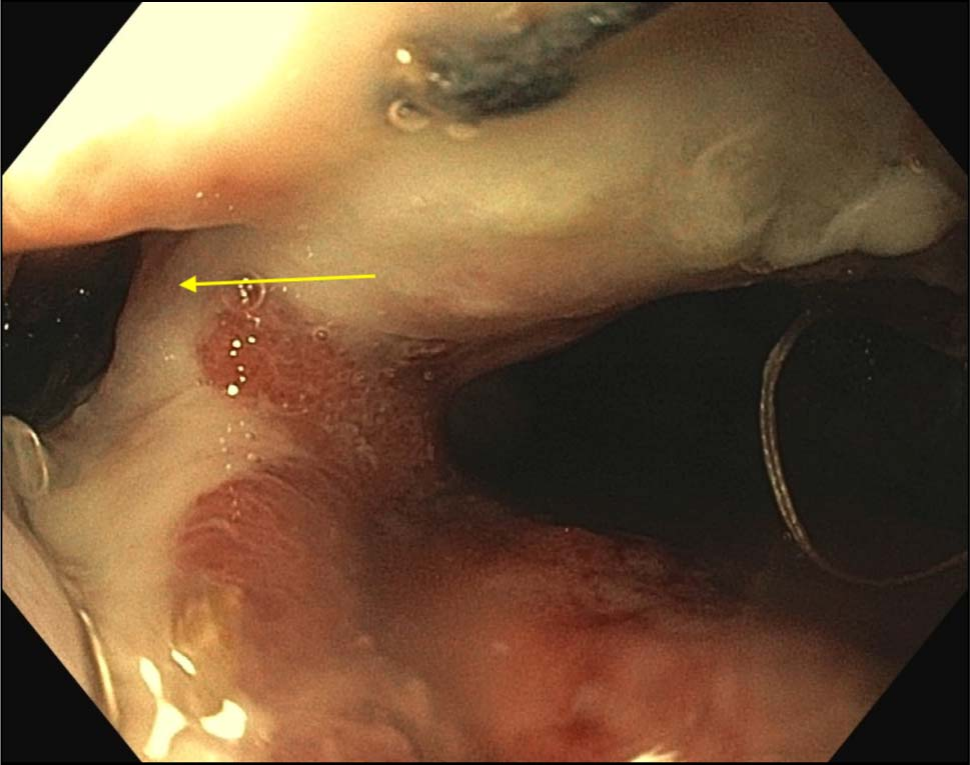
Novel endoscopic-vacuum therapy device in place adjacent to the defect.

**Figure 4.**
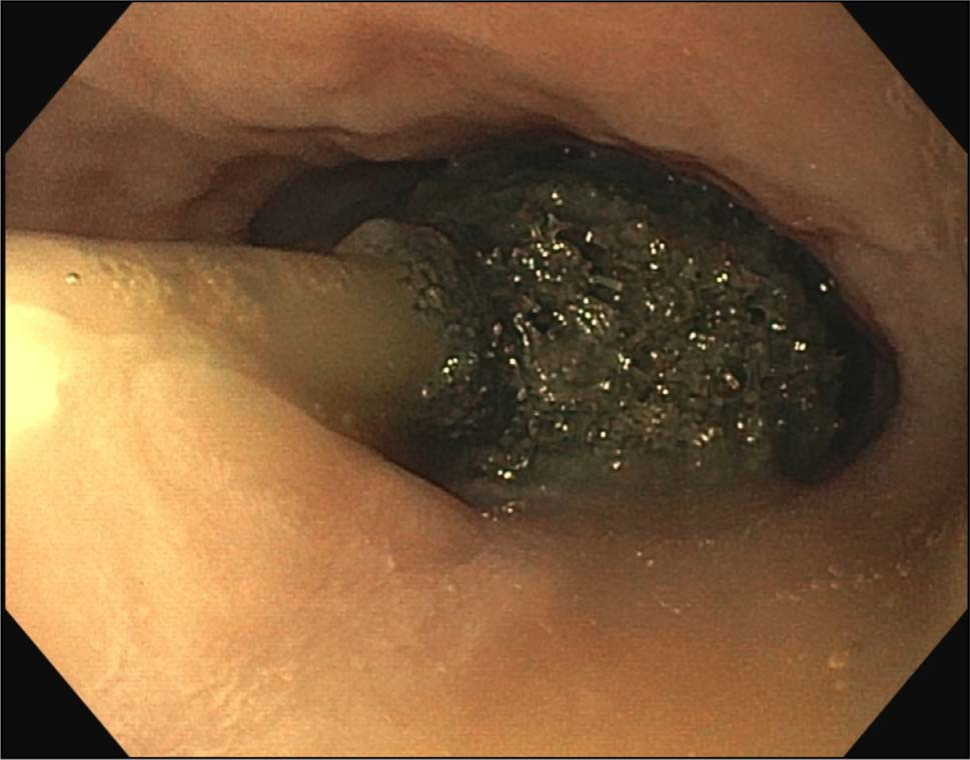
Novel endoscopic-vacuum therapy device after capsule has been dissolved, and sponge fully expanded.

**Figure 5.**
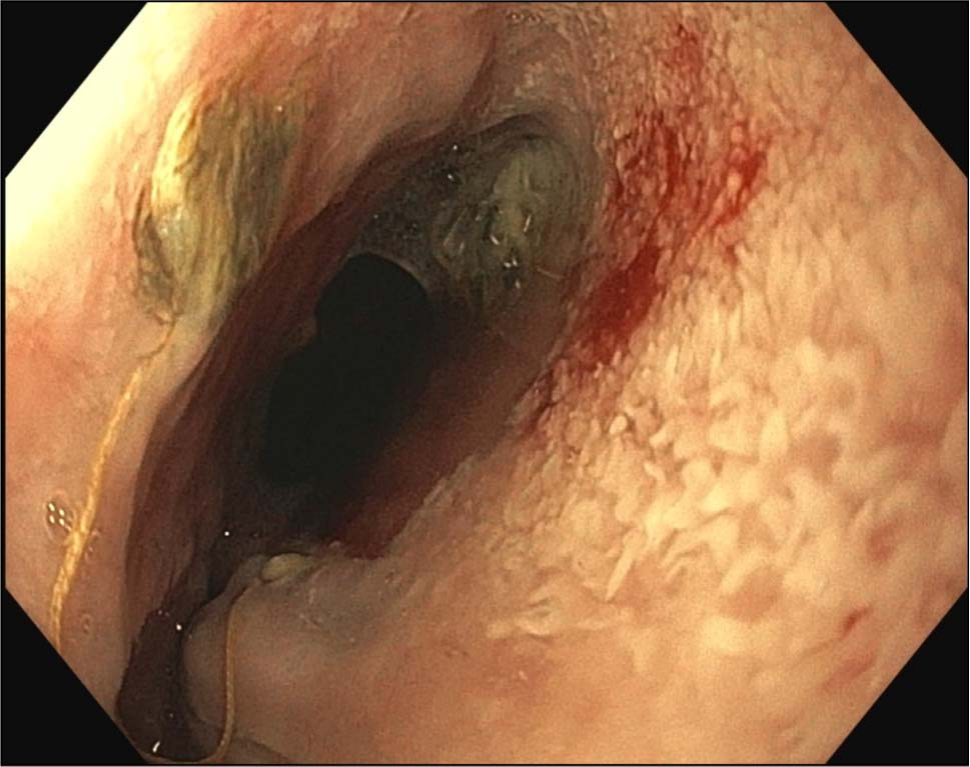
Granulation tissue formation and a decrease in size of the defect.

**Figure 6.**
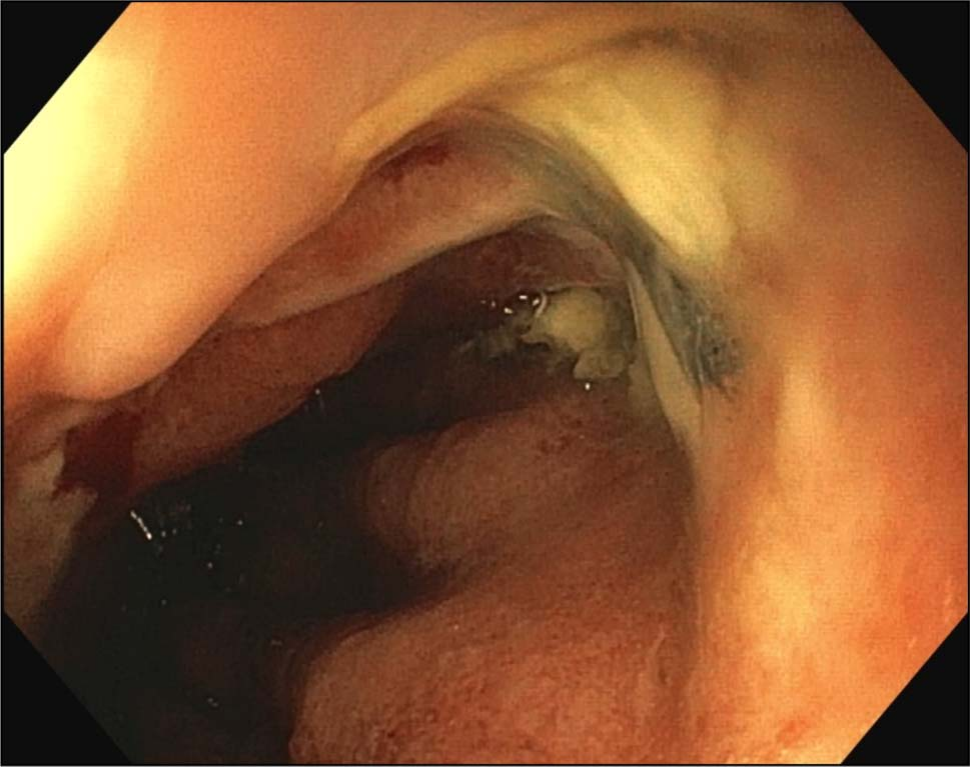
Complete resolution of the esophageal defect.

A 65-year-old man with long-segment Barrett's esophagus and esophageal adenocarcinoma underwent laparoscopic transhiatal esophagogastrostomy in December 2024. He presented to the emergency department 1-week after discharge for evaluation of sudden onset chest pain and hematemesis. Imaging revealed a large right-sided pleural effusion and a small pneumothorax, concerning for esophageal leak at the anastomotic site. Upper endoscopy revealed an esophago-gastric anastomosis 37 cm from the incisors, which was characterized by ulceration, an intact staple line, and an adjacent 9 mm defect (Figure 2). A novel one-piece EVT device was advanced through the nare into the esophagus, and positioned adjacent to the defect under direct endoscopic visualization (Figure 3). Sterile water was instilled to dissolve the degradable wrap and allow for full expansion of the sponge (Figure 4). The endoscope was withdrawn and the EVT device was attached to a portable wound-vacuum device (3M, San Antonio, TX) with pressure set to -125 mm Hg. The patient was kept nothing-by-mouth during the duration of EVT and received tube feeds via a previously placed jejunal feeding tube. The EVT device was exchanged under direct visualization with endoscopy every 4–5 days (3 exchanges total). Mean procedure time was 25.6 minutes (range: 24–30 minutes). Examination with each subsequent endoscopy revealed granulation tissue formation and a decrease in size of the defect (Figure 5). The final endoscopy performed on postoperative day 28 revealed complete resolution of the esophageal defect, and the EVT device was not replaced (Figure 6). Esophagram performed 1-day post-EVT removal was negative for persistent leak. There were no adverse events related to the novel EVT device. At 3-month, 6-month, and 1-year follow-up, the patient remained symptom free and was tolerating a regular oral diet. Surveillance endoscopy at 1 year showed well-healed mucosa without evidence of stricture.

## DISCUSSION

EVT is an innovative minimally invasive technique increasingly used in the management of gastrointestinal leaks, with a reported defect closure success rate between 81 and 88% and complication rate <10%.^2,3^ EVT has demonstrated superior outcomes in comparison to self-expanding metal stents, with a recent meta-analysis reporting a 21% increase in rate of defect closure, a 24% reduction in adverse events, and a 12% reduction in mortality.^4^ EVT works through the application of continuous negative pressure via an open-pore polyurethane sponge placed at the defect site, which promotes granulation tissue formation, enhances tissue perfusion around the defect, and provides continuous drainage which facilitates wound healing.

The conventional method for EVT involves passing a 10-Fr nasogastric (NG) tube (Cardinal Health, Dublin, OH) through the nare, grasping and removing the device from the mouth, and attaching the sponge to the NG tube. Before attaching the device to the NG tube, a commercially available wound vac sponge is cut to size (3M, San Antonio, TX), and a tunnel is created through the center allowing for placement of the NG tube through the sponge. A 2-0 proline suture (Covidien, Mansfield, MA) is used to fix the sponge to the proximal tip of the NG tube. A second suture is used to create a knotted loop at the tip of the NG tube, which allows for maneuvering the sponge into place across the defect with rat-tooth forceps under direct endoscopic visualization. This method is not without significant challenges, as achieving secure placement within the defect or against the leak site requires a high level of endoscopic skill, and inadequate fixation may lead to sponge migration or suboptimal therapeutic effect. In addition, variability in sponge material selection and construction methods can affect drainage efficiency, tissue contact, as well as overall durability.

Despite its demonstrated safety and efficacy, conventional EVT has faced barriers to widespread adoption related to the high level of skill required in placing the EVT device, and the technical complexity of device assembly. Intraprocedural assembly introduces variability in sponge size, shape, and attachment quality, potentially affecting treatment consistency across procedures. Furthermore, the learning curve associated with assembly and placement has limited EVT adoption to specialized centers with significant experience.^5^

This case describes the successful application of a novel EVT device, for the management of an esophageal anastomotic leak that occurred after esophagectomy. The design of this novel device addresses several recognized challenges associated with the conventional method, including eliminating the need for intraprocedural sponge assembly and has the benefit of reducing overall procedure time, anesthesia time, and minimizing variability in device configuration. The streamlined deployment with a degradable wrap allows for a slim profile that easily passes through the nares and improves device maneuverability. The flexibility of the device allows for deployment across right angles (intracavitary placement across the esophagus) and can be advanced over a wire to selected locations. This standardized design may facilitate broader adoption of EVT by reducing the technical learning curve and improving consistency across proceduralists with varying levels of EVT experience.^2,6^

The novel EVT device is available in a standard size which may limit adaptability to defects of various sizes, however in our clinical experience, 1 size fits most defects with an error to placing larger than needed sponges in cavities. The negative pressure results in collapse of the cavity wall and apposition of the extraluminal surface to the sponge.

Contraindications to EVT are not well defined in the literature; however, EVT therapy appears less effective for large (>6 cm) defects, chronic defects with established fistulas or tracts, defects associated with complex cavities or uncontained perforations, or cavities that cannot be accessed endoscopically.^7^ The novel EVT device is limited to nares that allow for passage of a 5 mm device. This can be relatively easily assessed by passage of an ultra slim scope (Olympus XP) or an 18F NG tube.

Some centers have successfully described using nasojejunal feeding tube placement along with vacuum therapy. This adds complexity to the procedure as fluoroscopy is generally required and either a double lumen tube (decreasing suction capacity) or separate tubes through each nare reducing patient comfort. Our facility has favored surgical jejunostomy tube placement for enteral feeding when EVT is anticipated to be of protracted duration. Further study is recommended to determine optimal feeding strategies to manage these complex and often protracted durations of therapy.

The favorable outcome and safety profile in this pilot study combined with the practical advantages of the one-piece device, suggests that pre-assembled EVT systems may help expand access to this effective therapy. As EVT demonstrates improved safety over stenting and surgical techniques, innovations that simplify the procedure and reduce variability will be essential for translating these outcomes beyond high-volume referral centers.

## DISCLOSURES

Author contributions: MA Cassera: study conception and design, data interpretation, drafting of the manuscript, critical revisions. L. Hilson, A. Singla, A. Baxi and M. Saunders: data interpretation, drafting of the manuscript, critical revisions. E. Seibel: study conception and design, drafting of the manuscript, critical revisions. AW Templeton: Guarantor of the article, study conception and design, data collection, data interpretation, drafting of the manuscript, critical revisions.

Financial disclosure: This project was supported by a Co-Motion Innovation Grant Award from the University of Washington, Seattle, Washington, USA. The funder(s) of this study had no role in study design, data collection, data analysis, data interpretation, or writing of the manuscript.

Previous presentation: This Brief Report was presented at the American College of Gastroenterology meeting in Phoenix, AZ on October 26, 2025. This presentation received the ACG Case Report Journal Award (Trainee) Category, and the ACG Presidential Abstract Award.

We have obtained IRB approval from the University of Washington to conduct this pilot study.

Informed consent was obtained for this case report.

## ABBREVIATIONS:

EVT: endoscopic vacuum-assisted therapy
NG: nasogastric
